# What service users with psychotic disorders want in a mental health crisis or relapse: thematic analysis of joint crisis plans

**DOI:** 10.1007/s00127-014-0869-1

**Published:** 2014-04-02

**Authors:** Simone Farrelly, Gill Brown, Diana Rose, Elizabeth Doherty, R. Claire Henderson, Max Birchwood, Max Marshall, Waquas Waheed, George Szmukler, Graham Thornicroft

**Affiliations:** 1 Section of Community Mental Health, Box PO29, Health Service and Population Research Department, Institute of Psychiatry, King’s College London, De Crespigny Park, London, SE5 8AF UK; 2 Division of Health and Wellbeing, Warwick Medical School, University of Warwick, Coventry, UK; 3 Division of Psychiatry, School of Medicine, University of Manchester, Manchester, UK; 4 Community Based Medicine, University of Manchester, Manchester, UK

**Keywords:** Choice, Shared decision making, Advance statements, Psychosis, Clinician–patient interaction, Crisis care

## Abstract

**Purpose:**

Recent legislation and guidance in England emphasises the importance of service user choice in care planning. However, it is not obvious how best to facilitate choices in care planning, and some clinicians are concerned that service users may make ‘unwise’ decisions. This study aimed to examine mental health service users’ preferences and priorities in the event of a future mental health crisis or relapse.

**Method:**

Thematic analysis of 221 joint crisis plans (JCP) developed by service users and their clinical team as part of the CRIMSON randomised controlled trial. Participants had a diagnosis of a psychotic disorder, at least one psychiatric admission in the past 2 years, contact with a community mental health team, and complex care needs.

**Results:**

Two major categories of preferences were identified: first the manner in which crisis care would be delivered; and second, specific treatment interventions. Most service users requested full involvement in decisions about their care, clear and consistent treatment plans, access to familiar clinicians who knew them well, and to be treated with respect and compassion. Some service users requested hospitalisation, but the majority preferred alternatives. The most frequently preferred intervention was care by a home treatment team. Just under half made a treatment refusal, the majority being for specific medications, alternatives were offered.

**Conclusions:**

Joint crisis planning resulted in service users making choices that were clinically reasonable. The technique employed by JCPs appeared to empower service users by engaging them in a productive dialogue with their clinicians.

## Introduction

Recent UK policy directives promote service user choice as one of the features defining a high quality health service, including mental health care [1]. NICE guidelines [2, 3] emphasise the need to consider service user treatment preferences and the Mental Capacity Act 2005 provides a legal framework by making formalised provisions for advance refusals of treatment [4]. Despite such guidance research suggests that in mental health care decision making remains dominated by clinicians [5–10].

‘Advance statements’ provide an avenue through which mental health service user choice may be facilitated. Advance statements allow individuals to make statements of preference regarding their future care at a time when they are well and have capacity to do so, but anticipating a time in the future when that capability is lost [11]. Advance statements differ in their legal enforceability between countries/regions and they may/may not involve clinical staff, family members or advocates in deciding the content. However, all types of advance statements have the goal of empowering service users and facilitating the expression of service users’ choices.

Potential benefits for advance statements in mental health care include: providing opportunities for service user empowerment [12, 13]; improving relationships between service users and clinicians [14–16]; and preventing future crises through careful planning [17]. However, service users have expressed doubts regarding the implementation of their statements [18], and studies suggest that clinicians are concerned that service users will refuse all treatment, or make choices counter to good practice guidelines [14, 19–22].

The joint crisis plan (JCP) [23, 24] is one form of advance statement. A difference between JCPs and other advance statements is the requirement for involvement of the mental health treatment team and an independent facilitator. While other advance statements such as the facilitated psychiatric advance directive (F-PAD) [15] do involve an independent person, and do sometimes involve clinicians, JCPs are the only type of advance statement that directly requires the involvement of the clinical team. The independent JCP facilitator ensures that all stakeholder views are obtained and the final content of the plan is the service user’s choice. The clinical team are present to discuss the plan and to help service users understand any limitations or barriers that they may envisage in implementing the plan, thus addressing several key concerns about advance statements mentioned above. The JCP therefore is a form of advance statement that encourages shared decision making, while emphasising the service user’s choice.

This paper describes a sub-study of the CRIMSON trial [16, 25]. The CRIMSON trial was a multi-site randomised controlled trial of JCPs compared with treatment as usual (TAU) for individuals with psychotic disorders. This sub-study analyses the content of JCPs to explore what types of requests service users make for crisis care. There have been few attempts to analyse the content of advance statements; however, analyses of Advance Directives in America [26] and the UK [27] suggest that service users’ preferences are clinically useful and consistent with practice standards. This paper seeks to extend this limited evidence base, to present service users’ preferences for crisis care in their own words and to examine whether joint crisis planning enables service users to develop clinically feasible crisis plans.

## Method

### The JCP intervention

The JCP [23, 24] contains the service user’s views on past treatments and preferences for care in the event of a future relapse/crisis. A series of content options (the JCP ‘Menu’—see Table 2 for headings) is presented to service users in an introductory meeting with the JCP facilitator. A second meeting is convened, a minimum of 1 week later, to finalise the content of the JCP. The minimum attendees at this second meeting are the service user, the JCP facilitator and the psychiatrist; however, the service user’s care co-ordinator, other relevant clinicians and family members were also invited.

The JCP facilitator chaired the meeting. Each item from the JCP menu was raised by the JCP facilitator, asking the service user if they would like to include that section on their JCP and if so, their ideas for content. The JCP facilitator would then ask other attendees if they had any comments regarding the proposed content, for example, the feasibility of being admitted to a preferred hospital/ward. The JCP facilitator would return to the service user and ask them what they would like entered under that section after considering the other attendees’ comments. It was the facilitator’s role to ensure transparent communication of everyone’s perspectives, and that service users did not feel pressured if there was a disagreement. The JCP facilitator would record the service user’s verbatim response for each section. The clinical team were asked at the end of the meeting whether they would agree to the content of the JCP. If they did not, the plan could still be completed (called a ‘crisis card’ and be held by the service user); however, this did not occur during the trial.

### Sample

CRIMSON ran in four mental health trusts in England including three cities and one rural area. Eligibility criteria were: diagnosis of a psychotic disorder; psychiatric admission in the last 2 years, and current contact with a community mental health team. Service users who were in hospital or under a section of the Mental Health Act at recruitment were not eligible to avoid any potential perceived coercion to participate. No other exclusions were made.

This sub-study reports on treatment preferences of CRIMSON participants randomised to the intervention group and who made a JCP. The trial received ethical approval by the King’s College Hospital Research Ethics Committee (07_H0808_174) and is registered with Current Controlled Trials ISRCTN11501328.

### Data collection

Demographic data including ethnicity [white, black (including Black African, Black Caribbean, Mixed Black-White and Black British) and other (mostly British Asian)], age, sex, marital status, diagnosis, number of hospitalisations were collected by interview and psychiatric medical records by research assistants.

### Data management and analysis

To ensure the representativeness of the sub-sample, comparisons were made (Chi-square/Wilcoxon-rank-sum tests) between those who did and those who did not complete a JCP on sex, age, marital status, ethnic group, diagnosis and number of admissions prior to baseline.

Using inductive thematic analysis [28], service user treatment preferences from JCPs were analysed. Analysis began by reading each of the JCPs. As the content of JCPs were direct quotes from service users and phrased in the first person, the data were considered akin to interview transcripts. A coding frame was developed inductively (that is based on data rather than pre-defined) and involved a random selection of 50 JCPs and coding each line of data. Where possible, codes used words directly from JCPs to keep the service users’ voice prominent in the analysis. Codes were then raised to a greater level of abstraction, by noting commonalities and differences between initial codes, using constant comparison. This initial draft of coding frame was discussed with the third author (DR—a service user researcher) and amended as necessary. The first and second author (SF and GB) then independently coded a further 20 JCPs. Ratings were compared and discrepancies discussed and resolved and the coding frame altered accordingly. SF and GB each coded half of the remaining JCPs using the revised coding frame. Superordinate categories were developed that captured common themes from across menu headings. In the results section, direct excerpts from JCPs are provided to illustrate the main themes. Any annotations from the authors (to improve readability and ensure anonymity) and JCP menu headings under which the data arose are provided in square brackets.

## Results

221 of the 285 (78 %) service users randomised to the intervention group made a JCP. The most common reasons for non-completion was refusal (*n* = 41), being too unwell (*n* = 9) or being discharged from services (*n* = 6). There were no differences between those who completed a JCP and those who did not in terms of sex, age, marital status, ethnic group, or diagnosis. However, those who did not complete a JCP had a slightly higher number of admissions in the 2 years prior to baseline (Wilcoxon-rank-sum test, *z* = 2.05, *p* = 0.04). The demographics of the sample that completed a JCP are shown in Table 1. The percentage of JCPs containing each optional heading from the JCP menu is shown in Table 2.

**Table 1:** Demographics of the sample (*n* = 221)

Variable	Category	Value
Age (years)	Mean (SD)	40.4 (1.44)
Sex	Male (%)	51
Ethnicity	White (%)	63.5
	Black (%)	23.5
	Other (%)	13
Diagnosis	Schizophrenia spectrum	74
	Affective psychosis	26
Marital status	Single (%)	58
Years in MHS	Mean (SD)	14.6 (9.4)

*MHS* mental health services

**Table 2:** Percent of JCPs containing each JCP menu option

JCP menu subheading	Frequency of inclusion
	*n* (%)
Nominee (person to I wish to be contacted in event of crisis)	184 (83)
Current care and treatment plan	
My mental health problem or diagnosis	219 (99)
Physical illnesses or allergies	122 (55)
Current care/treatment plan	207 (94)
Current medication and dosage	218 (99)
Circumstances that may lead to me becoming unwell or which have done so in the past	215 (97)
What happens when I start to become unwell	214 (97)
Treatments or other things that have been helpful during crisis or relapses in the past	205 (93)
Treatments or other things that have not been helpful during crisis or relapses in the past	151 (68)
Care in a crisis	
What I would like to be done when I start to become unwell	220 (100)
Preferred treatment or social care during a crisis or relapse	218 (99)
Specific refusals regarding treatment during a crisis or relapse	99 (44)
Circumstances in which I would wish to be admitted to hospital for treatment	171 (77)
Practical help in a crisis	
If I am admitted to hospital please contact the person named below and ask them if they would carry out the following tasks for me	132 (60)
If I am admitted to hospital I would like the following arrangements for my children/dependent relative	28 (13)
Other information I would like to be known or taken into account	69 (31)

The thematic analysis identified two major categories of responses in JCPs. The first category—‘delivery of care’—addresses the manner in which treatment was delivered and includes aspects of interpersonal interaction/communication and the availability of services. The second category describes the particular treatments/interventions that service users’ would/would not like in a crisis situation (e.g., medication and home treatment team).

### Delivery of care

Themes in this category referred to the manner in which clinicians interact with service users. There were four major themes: ‘Treat me with respect;’ ‘understanding what is illness, and what is not’; ‘continuity/consistency/clarity’; and ‘control and involvement’. Many of the examples from service users identified past experiences which they had found unhelpful and would not like repeated.

#### Treat me with respect

The wish to be respected was a fundamental theme in all the JCPs. Often respect was noted to be (or implied to be) lacking in the manner in which clinicians communicated. For example, being respected meant that clinicians took the time to explain and communicate their concerns and treatments they were considering:

“[Other information I would like to be known or taken into account] I would like people to voice or feedback to me symptoms they observe and tell me what’s wrong”.

“[Specific refusals regarding treatment during a crisis] I don’t want threats of an injection; I would like people to talk to me explaining the need to take medication”.

Respect could also be demonstrated by addressing service users’ experiences outside of symptom management and illness. For example,

“[Other information I would like to be known or taken into account] If I am in hospital for a long period I would like nurses to arrange for me to have a hair cut”.

Likewise, being prepared to be flexible in the aspects of delivery of care (e.g., consulting with service users regarding when home visits would be convenient) is another manner in which respect could be demonstrated:

“[Treatments or other things that have not been helpful in the past] The last time I was unwell, I felt Home Treatment Team messed me about. They came to my flat whenever it suited them. They wanted me to stay in all day. They wanted to visit me twice a day to give me my medication I couldn’t do that because I was in the middle of a divorce, I had appointments to see my solicitor, children and other commitments”.

#### Understanding what is ‘illness’ and what is not

Service users described situations in the past where clinicians/police have misconstrued their behaviour. One service user mentioned an experience of diagnostic overshadowing [29] when his physical illness was dismissed as a symptom of his mental health problem. Others described how appropriate expressions of emotion had been confused with symptoms of mental illness.

“[Treatments or other things that have not been helpful in the past] In the past the police have thought I was intimidating because I was ‘high’ (manic) and a tall black guy. I’m not intimidating, I can get agitated but not aggressive to others”.

Several service users highlighted how important it was for clinicians to know them as individuals to understand when a service user needs help. For example,

“[Preferred treatment or social care during a crisis or relapse] I have been in and out of hospital because the assessment was done by people who do not know me and didn’t pick up that I was becoming unwell so kept discharging me. I would like the Triage ward not to discharge me before speaking to my Consultant”.

#### Continuity, consistency and clarity

The majority of service users said that the initial contact when they started to feel unwell was their regular mental health team. Staff change was a source of additional stress for service users and often meant that there was a lack of continuity in their treatment:

“[Treatments that have not been helpful in the past] Staff changes. Treatments or help started by one member of staff on not being continued because of staff changes”.

One of the advantages of continuity of staff presented by service users was that the clinician would notice any change in their presentation and that the service user would trust them if the clinician told them that they were unwell. Many service users described feeling stressed when clinicians did not know that they had been involved in their care. When unwell, reducing novelty and increasing clarity regarding treatment plans helped to reduce the stress of relapse:

“[Treatments that have not been helpful in the past] Crisis team has been sending different people every day and changing medication too quickly”.

“[What I would like to be done when I first start to become unwell] Clarity with my medication—a proper plan of who is giving me my medication and when”.

#### Having control/involvement in decisions

Many service users identified a perceived lack of control over their mental life (e.g., communication or mood) as the first change when they start to become unwell. Many also described that the experience of crisis care can exacerbate these feelings of being out of control, for example, through irregular and inconsistent staff or a lack of clarity.

Service users described how it has been unhelpful in the past to be ‘bossed about’. The majority of service users expressed the wish to be involved in decisions about their care. For many, the desire to maintain some level of control was the reason for other treatment decisions such as wanting to be treated at home or admitted to hospital on a voluntary basis:

“[Preferred treatment or social care during a crisis or relapse] I would prefer to be in hospital on an informal basis so I can be involved in decision making around my care”.

A minority of service users identified that if they were to become unwell, they would prefer others (family or clinical team) to make decisions on their behalf.

### Specific treatments/strategies for dealing with crises

In this category, service users expressed preferences for specific treatments or strategies for crisis. Two themes involve non-medical intervention (e.g., self-management strategies; talking/support) and the others involved intervention from clinical staff. By far the most prevalent first preference for treatment in a crisis was for home treatment team support (35 % of the sample), followed by hospitalisation (19 %), and medication changes (14 %). For many, there was a clear preference for staging of these interventions, beginning at the non-interventional and ending, in serious cases, with hospitalisation:

“[Preferred treatment or social care during a crisis or relapse]. If I do relapse I agree to recommence on my depot (25 mg Risperidone Consta) and/or oral anti-psychotic medication (Risperidone). I would accept daily supervision of my medication by the Home Treatment team if necessary. If this fails I would be happy to come into hospital. I would prefer to do so informally”.

#### Self-management

For many service users, the first step in managing a potential relapse was to address their general health/wellbeing. Many described the need to reduce alcohol, to focus on eating well and to get enough sleep, relaxation and exercise. The importance of maintaining self care was also highlighted.

“[Treatments or other things that have been helpful in the past]… Activities such as tai chi, yoga and sports”.

“[Treatments or other things that have been helpful in the past] It is also very important for me to look after my appearance this makes me feel better”.

Most service users stated that they would do this on their own with their family’s help. In addition, while acknowledging the need to address a potential relapse, service users described the benefits of actively engaging in ‘normal’ aspects of life, such as work and relationships.

#### Talking and support

The majority of service users described the need for support and the opportunity to talk to someone about what they were experiencing to reduce the stress of the relapse. Like the theme of ‘understanding what is illness’, many service users highlighted the importance of clinicians’ understanding that they were experiencing difficult emotions.

“[Treatments or other things that have not been helpful in the past] Staff who have no respect or empathy for the fact that I am an adult who is suffering”.

Most often service users highlighted that it is helpful to talk to their regular clinicians, for example,

“[Treatments or other things that have been helpful during crises or relapses in the past]. Me telling staff how I feel. [My nurse] has helped me a lot by giving me good advice and helping me say ‘no’ to things. Seeing [my nurse] more regularly—once a week”.

Many service users also described the need for other supports such as friends and family being around to listen to their concerns. However, contact with family was not always straightforward for many service users who felt that family members may often get upset if relied on for support.

“[What I would like to be done when I first become unwell] I prefer not talking to someone who takes things personally (e.g. family)”.

#### Staying at home

For many service users, being able to stay at home for as long as possible was important. While 35 % of the sample described it as their preferred first line treatment the involvement of home treatment teams was amongst the preferences of 67 % of the sample. Staying at home provided service users enabled control of their experience and reduced the amount of disruption to their and their family’s life:

“[Preferred treatment or social care during a crisis or relapse] I prefer being treated at home because when I am in hospital I worry about my children”.

Some service users preferred to maintain contact with their regular team or care co-ordinator through home visits and to have extra support from the home treatment team if required. But by far the most common response to ‘preferred treatment or social care during a crisis or relapse’ was simply ‘the home treatment team’.

While home treatment for many was a simple proposition, for others the prospect of staying at home was dependent on having the family support. Some specified that if it became too much for their family, they would consider other alternatives:

“[Preferred treatment or social care during a crisis or relapse]. The Home Treatment Team can give me extra help. If the Respite home is available I could stay there. If [husband] is struggling I could come into hospital informally”.

While the majority of service users were positive about the home treatment team, three people stated that they would prefer not to have their involvement due to the anxiety associated with new people visiting. In these cases the service users stated they would prefer to go to hospital.

“[Preferred treatment or social care during a crisis or relapse]. I do not like strangers calling at the house so seeing the crisis team is not helpful, I would rather see the team I am used to and go into hospital if they felt I needed to”.

#### Medication

Just over half (56 %) of those who made a refusal, made a refusal about medication; 80 % of which related to a specific medication and often an alternative was presented. For example,

"[Specific refusals] Haloperidol I do not want, it makes me experience bad dreams. Risperdal makes me feel worse and I would prefer Olanzapine to Quetiapine".

The remaining medication-related refusals referred to injections, high doses and medication changes. Only one person stated that they would prefer not to take any medication. A far more common scenario was medication review/increase as a first strategy to deal with relapse; one for many service users that was preferable to hospitalisation.

“[Preferred treatment or social care during a crisis or relapse]. Review my medication small doses of zopiclone 3.75 mg preferably however initially I may have to have 7.5 mg to aid sleep, I would prefer zopiclone to diazepam. I would rather be seen by the HTT [home treatment team] than go into hospital”.

#### Hospital admissions

For the majority of service users, hospital admission was problematic. All had been admitted previously and most had strong views about the pros and cons of hospitalisation. For many, being taken to hospital and having to deal with new people and new settings were an additional stress to their relapse and could exacerbate the episode. Many described how hospitalisation did not help them feel better, but rather they felt bored, heavily medicated and trapped:

“[Circumstances in which I would wish to be admitted to hospital for treatment] In no circumstances would I agree with coming into hospital—it makes me more paranoid. There’s nothing they have in hospital that I need except for meds and I can take those at home. The only reason you get better in hospital is because you’re back on the meds and not because you’re in hospital”.

Eight percent (18/221) of the overall sample made a refusal in relation to hospitalisation, half of whom refused hospitalisation. The remaining refusals were associated with particular wards or being treated compulsorily. Most service users recognised that there will be situations when hospital admission is required and 77 % made a specific statement about when they would like to be admitted. Most preferred to go voluntarily to enable them to retain an element of control.

There were marked exceptions to this: several service users asked to be taken to hospital as soon as possible, believing that, based on prior experience, that they would deteriorate if left at home and it would prolong the episode.

“[What I would like to be done when I first start to become unwell] I become unwell and lose insight very quickly. If my family ring saying I’m ill or if I say I’m hearing voices then a bed must be found and if I won’t go in informally a warrant should be made immediately and the police must come and put me in hospital”.

## Discussion

This analysis indicates that service users with psychotic disorders make clinically reasonable requests for specific treatments in crisis/relapse situations. Two main categories of requests were found: the manner in which treatments were delivered, and specific treatment preferences.

The first category illustrated the importance of the manner in which interventions were or would be delivered. Aspects relating to involvement in decisions, having clear and consistent treatment plans, access to familiar clinicians, and being treated with respect were of clear importance to participants. These data indicate that current mental health service provision does not always achieve such individualised care and highlight some of the complexities involved in delivering mental health treatment. For example, an important component of good crisis care involves ensuring service users are able to access to services at all hours of the day, something one clinician cannot provide. Rather such provision may involve different members of the same clinical team and/or specialist teams, most of whom may not have been present during the discussion of the JCP. Thus there is a potential conflict between reliable access to help and ‘continuity/consistency/clarity’ requested by participants. Indeed one of the concerns of the clinicians in the CRIMSON trial is that while they may agree to the content of the plan, they may not be responsible for treatment in a crisis situation and therefore cannot ensure that the content will be followed [16]. This example highlights the need for transparent communication within JCP meetings.

The second major category of treatment preferences related to specific interventions. Consistent with literature [30], the most frequently preferred intervention was an alternative to hospitalisation, specifically home treatment team. Home treatment team care had the benefit of allowing service users to retain a sense of control and enabling participation in ‘normal’ activities. In contrast to clinicians’ concerns described in the literature [14, 19–22], less than half of the service users made a refusal and where there were refusals, valid alternatives were usually presented. Furthermore, while there were some refusals relating to medication and hospitalisation, these treatments were amongst the preferences for some service users, usually at specified stages of relapse. These analyses suggest that given an opportunity for involvement, the majority of service users would make requests for specific treatment that is currently being commissioned in standard care pathways in England. Furthermore, the opportunity to make a treatment refusal may provide clinicians with useful alternative treatment options in crisis situations.

This study may offer some tentative hypotheses regarding the lack of effect on involuntarily treatment in the CRIMSON trial. First, the clinicians were asked if they agreed to and could deliver the JCP and did so in all cases, yet some content may not always be achievable in routine care. Second, the proportion of treatment refusals was less in this study compared with 74 % in our previous trial [17]. There is no evidence to suggest that treatment standards have changed (e.g., involuntary treatment appears to be increasing [31]), making it reasonable to expect similar rates of treatment refusals. These two findings, combined with qualitative data [16] suggest the goal of transparent communication may not have been achieved, undermining clinician commitment to, and service user trust in the JCP. This is discussed further in papers currently under submission.

There are limitations to these data. While the presence of the Facilitator and clinicians is the strength of the JCP approach, it is possible that their involvement may have limited free expression of service users’ treatment preferences. The comparatively low proportion of refusals (i.e., 43 %) may underestimate the number of service users who might refuse treatment/make unfeasible requests. Similarly, while the Facilitator was present to empower service users, it is difficult to definitively alter existing communication patterns with one meeting. In this context, these data may overestimate the extent to which service users request interventions currently being delivered. Finally, considering the higher proportion of admissions in those who did not make a JCP this sample may under-represent individuals with more severe, relapsing conditions. The strengths to this study include: a large number of crisis plans from four geographical locations in England would suggest that these findings are likely to be generalisable; and the analysis provides clinically relevant service user preferences for approaches to crisis care and highlights the richness of information generated by this approach, compared to routine practice [6].

## Conclusions

The different treatment preferences of individuals with psychotic disorders highlight that an approach that generates the detailed perspectives of individuals should be sought. These analyses indicate that the manner in which crisis care is delivered is as important as the individual treatment strategies, and should be respectful, flexible and involve the service user as much as possible. The JCP provides a structured protocol to deliver these aims, while facilitating an equal and productive discussion between clinicians and service users.
